# Large‐scale whole‐exome sequencing association study identifies *FOXH1* gene and sphingolipid metabolism pathway influencing major depressive disorder

**DOI:** 10.1111/cns.13733

**Published:** 2021-10-11

**Authors:** Wei Zhou, Luan Chen, Bixuan Jiang, Yidan Sun, Mo Li, Hao Wu, Na Zhang, Xiaofang Sun, Shengying Qin

**Affiliations:** ^1^ Department of Obstetrics and Gynecology, Key Laboratory for Major Obstetric Diseases of Guangdong Province The Third Affiliated Hospital of Guangzhou Medical University Guangzhou Guangdong China; ^2^ Key Laboratory of Reproduction and Genetics of Guangdong Higher Education Institutes Guangzhou Guangdong China; ^3^ Key Laboratory for the Genetics of Developmental and Neuropsychiatric Disorders (Ministry of Education), Bio‐X Institutes Shanghai Jiao Tong University Shanghai China; ^4^ School of Life Sciences and Biotechnology Shanghai Jiao Tong University Shanghai China

**Keywords:** burden analysis, major depressive disorder, rare variants, UK biobank, whole‐exome sequencing

## Abstract

In the present study, we performed an exome‐wide investigation of the burden of rare disease‐causing variants for major depressive disorder (MDD) using 16,702 samples from UK biobank. Gene‐based association analysis and candidate gene prioritization analysis indicated that FOXH1 have significant association with MDD. In addition, sphingolipid metabolism pathway was found to be less enriched with rare disease‐causing variants in the MDD group, suggesting that this gene set may be involved in the pathophysiology of MDD.

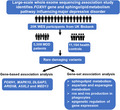

## CONFLICT OF INTEREST

The authors have no conflicts of interest to declare.

Major depressive disorder (MDD), the most common mental illness, is closely associated with physical and mental disability.^1^ Twin studies have shown that genetic factors are able to explain 30–40% of the variation in MDD.^2^ To date, genome‐wide association study (GWAS) has identified hundreds of susceptibility risk loci of MDD.^3^ However, studies of common genetic variations have estimated that the single genetic polymorphism (SNP)‐based heritability was only approximately 9–10%, reflecting a serious missing heritability problem.

Large sample size and precise description of clinical phenotype are two key points in identification of credible loci of psychiatric disease. However, strict clinical phenotypic inclusion criteria tend to limit the scale of sample collection. Several studies of rare variants based on whole‐exome sequencing (WES) with small sample size have been conducted to explore the risk loci of MDD.^4^, ^5^ However, the contribution of rare variants to the risk of MDD is not completely understood. Hence, a rare variant‐based association study with larger sample size and more strictly defined MDD is necessary.

In this study, we analyzed a cohort of 16,702 samples, including exomes data from 5,508 patients with MDD from UK biobank, which was released in October 2020. We defined and selected individuals who had both lifetime MDD and current MDD according to the descriptions of Cai et al.^6^ The basic characteristics of participants are shown in Data S1. Data acquisition was conducted based on the UK Biobank Application #34716. Written consent was acquired for all participants.

The protocol of WES production and quality control (QC) we used had been described in Van Hout et al.^7^ Protein‐altering single nucleotide rare (MAF <1%) variants (including missense, splice site, stop gain, start loss, and stop loss) were retained to assess whether there were significant enrichment differences in these variants through gene‐based and set‐based analysis. Weighted recursive truncated negative‐binomial regression (RUNNER), a novel gene‐based analysis, was also used to detect additional genes that were associated with risk of MDD.^8^ ToppGene, an online tool, was used to prioritize the significance of novel candidate genes from a reported MDD‐related genes list (Data S2). One thousand six hundred and four gene sets from Reactome V7.4 database and ten brain‐specific expression gene sets were selected to perform set‐based analysis. The threshold of significance of the association was defined as FDR *p* value = .05. Age was tested by means of the t test with normal distribution. Chi‐square test was performed to compare the frequency difference in sex, smoking status, usage of alcohol, and ethnicity between the MDD case group and controls by R version 4.1.0.

In this study, there were no significant differences of distribution in age and sex between MDD group (*N* = 5508) and control group (*N* = 11,194), as shown in Data S1. Fifteen genes were found to be significantly associated with risk of MDD (Data S3). We discovered and prioritized 7 candidate causal genes of MDD, which were *MAPK10*, *FOXH1*, *DLGAP3*, *ARID5B*, *ASXL2*, and *MED13* (Table 1). We also found that 4 gene sets were significantly associated with MDD (Table 2). In addition, the top gene set was found to be involved with sphingolipid metabolism (FDR *p* value = 1.76 × 10^−4^). Remarkably, the synonymous variants in genes from the significant gene set mentioned above did not appear to have significant association with MDD (Data S4).

**TABLE 1:** Results of the candidate genes prioritized by ToppGene

Rank	GeneSymbol	GO: Molecular function score	GO: Molecular function pValue	GO: Biological process score	GO: Biological process pValue	GO: Cellular component score	GO: Cellular component pValue	Disease score	Disease pValue	Average score	Overall pValue
1	*MAPK10*	0	0.507746	0.995069	0.044733	0.971393	0.00639	0.437813	0.075136	0.496733	1.1E−05
2	*MEOX2*	0.598343	0.00213	0.969953	0.063129	0.559724	0.021301	0.738734	0.048799	0.437423	0.000405
3	*FOXH1*	0.598343	0.00213	0.997541	0.039117	0.277442	0.031952	0	0.54938	0.351622	0.007687
4	*DLGAP3*	0	0.507746	0.99767	0.038923	0.995807	0.002905	0.970353	0.023044	0.399189	0.009506
5	*ARID5B*	0	0.507746	0.996831	0.040279	0	0.525562	0.990815	0.018203	0.37592	0.014052
6	*ASXL2*	0.340202	0.006778	0.999893	0.023431	0	0.525562	0.234146	0.093726	0.315989	0.017739
7	*MED13*	0	0.507746	0.868756	0.084431	0	0.525562	0.761332	0.047444	0.31631	0.046889
8	*SLC11A1*	0	0.507746	0.999993	0.017622	0	0.525562	0.42769	0.07591	0.261259	0.063128
9	*CEP63*	0	0.507746	0.752722	0.098954	0	0.525562	0.852241	0.039117	0.340742	0.066611
10	*NUP153*	0.340202	0.006778	0.480719	0.12103	0	0.525562	0	0.54938	0.25283	0.073663
11	*NBEAL2*	0	0.507746	0.798979	0.095081	0	0.525562	0.241872	0.09237	0.243564	0.109382
12	*PITPNM3*	0	0.507746	0	0.573199	0.606883	0.021301	0	0.54938	0.179497	0.198996
13	*ZNF469*	0	0.507746	0	0.573199	0	0.525562	0.925116	0.029047	0.187158	0.232404
14	*BRPF3*	0	0.507746	0.685318	0.1067	0	0.525562	−1	0	0.193478	0.351954
15	*KIFC2*	0	0.507746	0	0.573199	0	0.525562	−1	0	0.184741	0.574996
16	*KIAA1522*	0	0.507746	0	0.573199	0	0.525562	0	0.54938	0.117213	0.578612

**TABLE 2:** Significant set‐based association result of rare (MAF <1%) damaging non‐synonymous variants from reactome database

GeneSet	No. of variants	CaseAltAlleles	ControlAltAlleles	skato	skatofdr
Sphingolipid metabolism	58	318	602	9.65E−07	0.000176
Aspartate and asparagine metabolism	3	33	133	0.000306	0.018544
ROS and RNS production in phagocytes	26	267	521	0.000279	0.018544
Epigenetic regulation of gene expression	50	516	937	0.000762	0.034681

Notes

skato: optimized sequence kernel association test.

*FOXH1* was a candidate risk gene of MDD that was identified by two burden analysis and candidate gene prioritization analysis in this study. *FOXH1* encodes xenopus forkhead activin signal transducer‐1 and is highly expressed in the brain. *FOXH1* plays an important role in TGF‐beta signaling pathways. It has been reported that TGF‐beta pathways modulated psychiatric disorders.^9^ Further, our results were in consistence with previous studies, which have shown that there were significant correlations between changes in sphingolipid metabolism and anxiety‐like behavior in female rates.^10^ Moreover, the result of set‐based analysis showed that genes in hypothalamus region were associated with MDD. In fact, hypothalamic‐pituitary‐adrenal (HPA) axis abnormalities play an important role in the associations of MDD risk.^11^

In conclusion, our study identified several candidate risk genes of MDD and the sphingolipid metabolism pathways were associated with MDD. While the anatomic substrate in MDD remains unclear, our findings provide important insight into the molecular basis of MDD.

## Supporting information

Data S1

Data S2

Data S3

Data S4

## Data Availability

All data are available from UK biobank database.
